# Is frailty a predictor of mortality in late-life depression?

**DOI:** 10.1192/j.eurpsy.2022.455

**Published:** 2022-09-01

**Authors:** M. Arts

**Affiliations:** GGZWNB, Psychiatry, Halsteren, Netherlands

**Keywords:** Depression, Frailty, mortality

## Abstract

**Introduction:**

Frailty is a clinical phenotype that predicts negative health outcomes including mortality. Similar to frailty, late-life depression is also associated with increased mortality rates.

**Objectives:**

Our objective was to examine whether frailty and frailty related biomarkers predict mortality among depressed older patients.

**Methods:**

Among 378 older patients (≥60 years) with a depressive disorder (DSM-IV criteria) we examined whether frailty predicts time-to-death during a six-year follow-up using Cox-regression analyses adjusted for confounders. Baseline data were collected between 2007 and September 2010. Frailty was defined according to Fried’s criteria (muscle weakness, slowness, exhaustion, low activity level, unintended weight loss). Similarly, we examined the predictive value of three inflammatory markers, vitamin D level, and leucocyte telomere length, and whether these effects were independent of the frailty phenotype.

**Results:**

During follow-up, 26.2% frail depressed patients died compared to 12.7% non-frail depressed patients (p<.001). Adjusted for confounders, the number of frailty components was associated with an increased mortality rate (HR=1.38 [95%CI: 1.06–1.78], p=.015). All biomarkers were prospectively associated with mortality, but only higher levels of hsCRP and lower levels of vitamin D were independent of frailty associated with mortality.

**Conclusions:**

Frailty identifies older patients at increased risk of adverse negative health outcomes in late-life depression. Therefore, among frail-depressed patients, treatment models that include frailty-specific interventions might reduce mortality rates.

**Disclosure:**

No significant relationships.

